# Covert observation in practice: lessons from the evaluation of the prohibition of smoking in public places in Scotland

**DOI:** 10.1186/1471-2458-7-204

**Published:** 2007-08-10

**Authors:** Mark Petticrew, Sean Semple, Shona Hilton, Kaen S Creely, Douglas Eadie, Deborah Ritchie, Catherine Ferrell, Yvette Christopher, Fintan Hurley

**Affiliations:** 1 MRC Social and Public Health Sciences Unit, Glasgow, UK; 2 Department of Environmental & Occupational Medicine, University of Aberdeen, UK; 3 Institute of Occupational Medicine, Riccarton, Edinburgh, UK; 4 Institute for Social Marketing, University of Stirling and Open University Dept of Marketing, University of Stirling, Stirling, UK; 5 University of Edinburgh School of Health in Social Science, Edinburgh, UK

## Abstract

**Background:**

A ban on smoking in wholly or substantially enclosed public places has been in place in Scotland since 26^th ^March 2006. The impact of this legislation is currently being evaluated in seven studies, three of which involve direct observation of smoking in bars and other enclosed public places. While the ethical issues around covert observation have been widely discussed there is little practical guidance on the conduct of such research. A workshop was therefore convened to identify practical lessons learned so far from the Scottish evaluation.

**Methods:**

We convened a workshop involving researchers from the three studies which used direct observation. In addition, one of the fieldwork managers collected written feedback on the fieldwork, identifying problems that arose in the field and some solutions.

**Results:**

There were four main themes identified: (i) the difficulty of achieving and maintaining concealment; (ii) the experience of being an observer; (iii) the risk of bias in the observations and (iv) issues around training and recruitment. These are discussed.

**Conclusion:**

Collecting covert observational data poses unique practical challenges, in particular in relation to the health and safety of the researcher. The findings and solutions presented in this paper will be of value to researchers designing similar studies.

## Background

Covert research and the use of covert methods have always been contentious, on the grounds that they may involve the deliberate misleading of the public or other study participants [1]. However, they offer researchers access to information that is otherwise unavailable, and from a scientific perspective they offer the prospect of collecting objective data while minimising Hawthorne effects and other biases [2,3]. Social desirability bias, for example, has been shown to particularly affect the measurement of health behaviours, including self-reports of diet, smoking and alcohol consumption, physical activity and sexual behaviour [4-6].

Covert observation is one approach to the problem of collecting such behavioural data, and it has attracted a considerable literature. One of the most well-known examples is sociologist Laud Humphreys' study of the "Tearoom Trade" in the United States [7]. Humphreys observed hundreds of sex acts between men in public toilets while acting as the "watchqueen" who does not take part but watches out for the police. The degree of deception involved led to much public and academic criticism and the study is still used as a teaching example on ethics courses.

Other studies have used similar approaches, for example involving covert participation by researchers in gangs and religious cults [8,9]. At the other end of the scale, however, is the covert observation of behaviour in public places without actually taking part. Such non-participative approaches to data collection may be more acceptable if the recording of the behaviour does not have negative consequences for those observed [10]. Examples in clinical settings include covert, non-participative observation of handwashing [11] and child behaviour [12], and it has also been used to assess the effects of workers' behaviour on their exposure to chemical hazards [13]. In such situations the researcher cannot be accused of encouraging or participating in the activities they are recording, but instead acts as a "fly-on-the-wall": observing and recording events while seeking to avoid influencing their occurrence.

The challenges to covert observational work came under consideration during the evaluation of the Scottish ban on smoking in enclosed public places which was implemented in Spring 2006. Three of the seven studies currently evaluating the health impacts of this legislation involve direct observation of smoking in bars and other public places. The legislation and the evaluation (See Table 1) are described briefly below. The evaluation is being carried out by the CLEAN ("Clean-Air Legislation Evaluation") Collaboration, a group of researchers in Scotland carrying out a series of linked evaluation studies which address specific research questions relating to the impacts of the ban (see Appendix).

**Table 1:** Summary of research commissioned to evaluate the impact of the Scottish Smoking ban [14].

**Study**	**Aim, Design & Data Collection**
**Changes in Ch**ild Exposure to Environmental **T**obacco **S**moke (**CHETS**)	**Aim: **To determine change in childhood exposure to ETS, including exposure in the home. **Design**: Repeat cross-sectional survey of probability sample of Scottish Primary 7 children (11 yrs). **Sample size: **2,500 children pre and 2,500 post ban **Data collection**: Baseline Jan-Feb 06; follow-up Jan-Feb 07. Self-complete questionnaire on smoking status and self-reported exposure; saliva sample (for cotinine assay).
**H**ealth **E**ducation **P**opulation **S**tudy (**HEPS**)	**Aim: **To determine change in adult exposure (non-smokers) to ETS in the home and public places and changes in tobacco consumption (smokers). **Design**: Repeat cross-sectional in-home survey of probability sample of Scottish adults (16–74 yrs). **Sample size: **1,800 adults pre and 1,800 post ban **Data collection**: Baseline Sept-Oct 05/Feb-Mar 06; follow-up Sept-Oct 06/Feb-Mar 07. Interviewer-administered questionnaire on smoking status, self-reported exposure and attitudes towards smoking and legislation; saliva sample (for cotinine assay).
**ST**udy **O**f **P**ublic place **I**ntervention on **T**obacco exposure (**STOPIT**)	**Aim: **To determine change in the incidence of acute coronary syndrome **Design**: Multi-centre prospective study of hospital admissions for acute coronary syndrome. Entry criteria: chest pain + raised troponin on admission/within 12 hours. **Sample size: **ACS cases from 9 centre, representing half Scottish ACS admissions (circa 2,700 per annum) **Data collection**: Continuous May 05-Apr 07. Research nurse-administered questionnaire on smoking status and self-reported exposure; admission blood sample (for cotinine assay).
**B**ar-workers' **H**ealth and **E**nvironmental **T**obacco **S**moke **E**xposure (**BHETSE**) *	**Aim: **To determine change in respiratory health of bar workers **Design**: Prospective cohort study of bar workers from five urban and rural areas in Scotland. **Sample size: **371 bar workers recruited at baseline and followed up; direct measurement of air quality in 41 bars. **Data collection**: Baseline Jan-Feb 06; follow-up Jun-Jul 06 and Jan-Feb 07. Interviewer-administered questionnaire on smoking status, self-reported exposure and attitudes towards smoking and legislation; lung function (FEV_1_, FVC); saliva sample (for cotinine assay). Air sampling for PM2.5 in selected premises.
Qualitative Bar Study *	**Aim: **To determine changes in attitudes and behaviour in relation to smoking, smoking restrictions and the cultural contexts in which smoking and drinking take place. **Design: **Qualitative pre- and post- study of bars and their customers in three communities. **Data collection: **In-depth and paired interviews, direct observation.
Qualitative Community Study *	**Aim: **To determine impact of legislation on attitudes, behaviours and experiences of individuals, families and communities. **Design: **Qualitative pre- and post- study of four contrasting communities. Nested case study approach. **Data collection: **Baseline Sept 05-Mar 06, follow-up Apr-Dec 06. In-depth interviews, focus groups, direct observation of enclosed and outdoor spaces.
**I**nternational **T**obacco **C**ontrol (**ITC**) Ireland/UK Scotland Extension	**Aim: **To determine changes in smokers and non-smokers attitudinal and behavioural response to smoke-free laws **Design: **Quasi-experimental prospective cohort telephone survey of probability samples of smoking and non-smoking adults in Scotland, the rest of the UK and Ireland **Sample size: **500 smokers and 300 non-smokers at baseline. **Data collection**: Baseline: Feb-Mar 06; Follow-up Feb-Mar 07. Telephone survey on smoking status; quit attempts in smokers; attitudes towards and compliance with legislation; social norms about smoking, smoking behaviour in public and private venues

* Study includes covert observational research

### The Smoking Ban, and the CLEAN framework

A ban on smoking in wholly or substantially enclosed public places came into force in Scotland on 26^th ^March 2006. The legislation makes it an offence to smoke, or to permit smoking in such premises. To evaluate the impacts of the legislation Scotland's health improvement agency, Health Scotland, and the Scottish Executive (Scotland's devolved government) developed an evaluation framework involving new data collection for up to one year and routine data for up to 3 years after the legislation was introduced. The full evaluation framework is described elsewhere [14].

As part of this framework, two qualitative studies (the *Qualitative Bar Study*, and the *Community Study*) were commissioned. Both studies use qualitative methods including in-depth interviews, focus group discussions and observation.

*The Qualitative Bar Study *examined changes in attitudes and smoking behaviour among bar customers and bar workers and assessed changes in the cultural contexts in which smoking and drinking takes place. The study collected data from a cohort of bar customers, bar workers and bar managers from selected premises both before and after implementation of the smoke-free legislation. In-depth interviews were conducted, along with five one-hour observations in each of the selected premises. All of these observations were conducted by a middle-aged, male observer from outside the study area who matched the broad customer profile of the study bars. Data collection was restricted to non-participant observation and information was also collected on the numbers and profile of staff and customers (including smoking behaviour, visibility of tobacco products and levels of compliance), the provision of ashtrays, the existence of outside smoking facilities, signage, ventilation, and the sale of tobacco and related products. Observations were recorded on a semi-structured pro-forma and were conducted at peak drinking times and on the same day and time to aid observer anonymity and comparability of data.

The *Qualitative Community Study *examined the broader impact of the legislation at individual, family and community levels in two contrasting local authority areas, one urban and one semi-rural. The aim was to explore the social context and impact of the smoking legislation on individuals, families, community and public spaces.

Systematic observations were conducted within each area in order to observe the social practices within different social contexts of smoking and non-smoking during the 3–6 months before the legislation came into force, immediately after implementation and 6–9 months post-implementation. The locations were identified as 'typical' (e.g., bars, bookmakers, lunch clubs and other community facilities) of the areas. Each location was sampled at various points in time and across the study period to ensure that time-of-day and seasonality effects were captured. Observational templates were developed in order to collect data on key indicators, such as the type of the place and the use of the space, the characteristics and behaviours of the smokers/non-smokers, signage, ashtrays and positioning of smoking materials, and any contraventions of the ban.

The observational element of the *Qualitative Community Study *presented a number of challenges, which were overcome primarily by working within existing community networks. Where there were particular concerns for safety, such as in areas of socio-economic deprivation, local people were recruited through local community projects or contacts to accompany the fieldworker into the location. Two female fieldworkers were deployed in each location to act as participant observers. Blending into the context aimed to reduce the risk of threats to personal safety and to limit any bias introduced through observer effects.

There are ethical issues involved in 'covert' observation in a community context, in particular the potential to violate the principle of informed consent and the need to avoid invading personal privacy [15]. However, all the places in which data collection occurred were 'public places' and the individuals and the specific locations and individuals remain protected by anonymity and confidentiality. Personal information concerning research participants that may have been inadvertently divulged during the observations and through the unavoidable conversations that occur has been kept confidential and under review to identify any sensitive material that may not have been appropriate to record. (see table 2)

A further study, the *BHETSE study *(Bar workers Health and Environmental Tobacco Smoke Exposure) followed a cohort of bar workers with the aim of assessing changes in their environmental tobacco smoke (ETS) exposure and self-reported respiratory symptoms before and after the implementation of the ban. The *BHETSE study *proposal was submitted for ethical review to the Grampian Research Ethics Committees – NHS Grampian and Aberdeen University. The chairman of the ethics committee reviewed the complete project proposal and stated that both the LREC and University of Aberdeen Ethics Committee did not require ethical approval to be sought. All studies carried out by investigators based at the University of Aberdeen, Institute of Applied Health Sciences, must adhere to the Research Governance policy and in accordance an ethical review monitoring process was set up for *BHETSE*. This process was part of the remit of the project Advisory Committee Group established by NHS Health Scotland. The committee met and considered the ethical aspects of the study on several occasions prior to field work commencing.

As part of the *BHETSE study*, measures of particulate matter of less than 2.5 microns (PM_2.5_), an air-marker of ETS exposure, were also made in selected premises. *BHETSE *also aims to test associations between reduced ETS exposure and objective measurements of lung function. Part of the *BHETSE study *also involved covert 30-minute visits to bars in Glasgow, Aberdeen and Edinburgh during busy and quiet periods both before and after the implementation of the ban. Data on bar characteristics (e.g., size, ventilation, food sales), customers (including overall numbers, and numbers of smokers), and bar staff (smoking behaviour) were collected by researchers who visited the bar to collect this information. During their visit observations were made and data were recorded on pro-forma worksheets when they had left the premises. In some of these visits the researchers also undertook covert measurement of PM_2.5 _levels using a small monitor concealed in a shoulder bag. Full details of the methods are given elsewhere [16].

### Previous observational work on the impact of smoking restrictions

Observational work has previously been carried out as part of evaluations of the impact of smoking restrictions. Sites of previous studies include hospitals [17-20], workplaces [21,22], shops [23] and supermarkets [24], elevators [24], and cafeterias [25]. However, these studies do not discuss in detail the challenges associated with covert observation. These challenges fall into two main categories: (i) the difficulty of achieving and maintaining concealment, and (ii) the ethics of concealment. As noted above, there has been extensive discussion of the ethical issues of observational work over the past 4 decades [1,26,27] and guidelines have been issued by professional societies such as the Social Research Association [28]. However, the practical challenges have been much less well-described. This paper therefore presents a summary of the practical issues arising from covert observations conducted as part of an evaluation of the Scottish Smokfree legislation.

## Methods

We convened a workshop at the Institute of Occupational Medicine in Edinburgh on 13^th ^June 2006 involving researchers who had been involved in collecting observational data as part of the Scottish evaluation. The workshop was convened to discuss the practical, ethical and other problems which the work had uncovered, and to discuss solutions that had been developed by the respective research teams. In addition, one of the fieldwork managers (CF) asked researchers involved in data collection in the *BHETSE study *for written feedback on the fieldwork. Notes of the discussions were taken by two participants (MP, SS), and information from these sources is summarised below, grouped under four broad headings: (i) the difficulty of achieving and maintaining concealment; (ii) the experience of being an observer; (iii) bias in the observations and (iv) training and recruitment.

## Results

### (i) The difficulty of achieving and maintaining concealment

The difficulty of "fitting into" a bar they had never previously visited and of discreetly observing smoking was probably the main concern for the researchers who collected these data. The ability to "blend in" depended on both the type of bar and the time of day. Matching the observer by age and gender to the bar was important in some circumstances, so that they looked like a "typical customer". In this respect busy city centre pubs were easier venues in which to collect data than "local" bars with fewer customers, most of whom would have been well-known to each other and to the bar staff. One solution when collecting data in a small community on the other hand was for the researcher to spend the day in the area in order to become "known". Even then a researcher would sometimes have attracted notice and one of the research teams employed a local person to accompany the observer. For the same reason, it was considered that it was sometimes better to visit in a group rather than as an individual.

In bars where data on air quality were also being collected by means of a portable aerosol monitor the time of day was also important. Background bar noise helped mask the noise of the monitor; the noise seemed more obvious in daytime in a quiet bar. The need to have a shoulder bag to conceal the monitor also posed some difficulties, as there were venues where the bag seemed out of place. However, no security or bar staff questioned the contents of the bag or prevented the researchers from entering any premises.

Collecting data on smoking could also be difficult, given that it would have been obtrusive and out of place for the observer to sit in the bar and fill in the data collection forms. All teams were therefore under strict instructions not to take questionnaires or similar documentation into the venues. Whilst it was possible to make discreet notes (for example, on a newspaper, crossword, Sudoku grid or mobile phone) during the day, this would usually have seemed out of place at night. One solution was to visit the bar in pairs, having memorised the protocol, with the data collection then being shared by two observers. Taking photos by mobile phone was generally not possible for safety reasons. However, several of the researchers sent voicemails to themselves describing their observations so that the data could later be transcribed.

Inadvertent violations of bar norms were also possible; a researcher could accidentally sit in a seat belonging to a regular customer. However, the most obvious measure the researchers could take to "fit in" was to have a drink in the bar. Sometimes a soft drink was possible; but in some bars at some times this, too, could attract attention. In one venue putting money in the jukebox was also found to constitute unusual behaviour.

Despite taking precautions, the purpose of the researchers was on occasion almost exposed. In one case the bar manager asked why they were writing on their newspaper; they made an excuse and left the bar. On another occasion one of the bar staff knew a friend of one of the researchers. As a result of these experiences the researchers emphasised the importance of having a previously-rehearsed reason for being in the bar. While the practice of maintaining concealment may appear unethical, it is also a simple necessity if the health and safety of the fieldworker is to be protected. Researchers did not proactively go out of their way to appear to be "one of the regulars", (e.g., deliberately mixing and socializing with customers) but instead took necessary measures not to attract attention. When they thought that they were doing so they withdrew as described. Nor did this happen often enough to prejudice the quality of the data (in two bars only, out of over 70 bars in these studies).

The use of some bars for illegal activity, particularly drug dealing, may also pose particular problems. In addition to the direct risks to personal safety and the possibility of loss of expensive monitoring equipment (each personal aerosol monitor costing in excess of £1500), there are issues relating to the clientele being sensitive to the possibility of the presence of under-cover police officers. This was further compounded during visits post-ban where many bars were aware that environmental health officers were undertaking spot checks to check for contraventions of the new regulations.

### (ii) The experience of being an observer

There was undoubtedly an impact on the researchers themselves. Researchers often reported feeling somewhat paranoid, partly because of risk of being recognised, but also in cases where the noise of the air sampler seemed obtrusive in a quiet bar, though this could be masked. The air sampler was not used in bars where the risk was deemed unacceptable.

Within the aims of these studies the health and safety of the researchers was paramount, and fieldworkers were told to leave the bar or not to enter if there was felt to be an unacceptable risk. Risk assessments were prepared in advance and "Lone worker" protocols were also used. This is a procedure for monitoring the safety of lone workers, which allows them to call for immediate assistance [29].

One further problem was that researchers sometimes felt a conflict of interest between collecting the data and observing criminal activity – particularly drug dealing in pub toilets. One key message from the fieldwork was that researchers should not visit the toilets in bars where this was a risk. They were also advised that if they were to see an act committed they were in the same position as any other member of the public; they were not to attempt to prevent the act taking place if it involved property, but that it should be a personal decision if it involved the harming of a third person, and they should consider calling for help. Researchers also reported removing jewellery, handbags or other valuables to avoid attracting attention or risking having them stolen.

### (iii) Bias in the observations

Observation bias is well-documented in many other scientific fields. It refers to the systematic biases inadvertently introduced when observing events (or behaviours). Researchers were conscious of this bias, but pilot work suggested that the use of a protocol and standardised pro-formas reduced the risk, and there was generally high agreement between the data collected by two observers in the same bar. Using two observers in the field also limited such biases.

One other possible bias is selection bias in the sampling of bars. In Glasgow two bars were excluded as the risk to researchers was deemed to be too high. However, this will probably have little impact on the generalisability of the study findings. Bars from the poorest areas are well-represented, as are bars which presented some potential risk.

### (iv) Training and recruitment

Researchers collecting the data for the *BHETSE study *took part in a two-hour training session covering data collection and recording. In retrospect additional training involving role-playing and perhaps visits to bars at times closer to the times when they would have been collecting data would also have been useful. The researchers themselves had a range of previous experience, and in the case of the *BHETSE study*, the survey manager who dealt with the recruitment and training felt that it was important to choose observers on the basis of self-confidence, previous experience, and the ability to "fit into" the environment; this, coupled with a thorough grounding in social science methods and good practice, would probably be sufficient for most studies.

## Discussion

These data provide a novel methodological contribution to ongoing debates about observational methods of data collection. The findings may be widely applicable even though they were collected in the context of an evaluation of a smoking ban. They may also be particularly relevant to the evaluations of smoking bans that are being planned or proposed in other countries.

It is difficult to draw parallels with previous research as similar studies have not often reported methodological issues in detail. Previous studies of covert participation in social groups, (e.g., gangs, neo-Nazi groups, or bars [9] and the extensive deception this may involve are very different from the non-participatory observation of legal behaviour in public places, as described in this paper, which it has been argued are relatively free of ethical problems [30]. Nonetheless there remains a need for critical reflection on the meaning and value of the data collected and on the balance between the social value of the data and the possibility of harm to participants arising from the potential deception inherent in this type of work [9]. Harm to researchers is another possibility. The risks to personal safety from being discovered are discussed above, but where data were collected in bars there is also a need to consider the health effects of exposure environmental tobacco smoke. The benefits of using covert methods on the other hand include the ability to collect unbiased data, which would in turn shed light on the effectiveness of an important policy intervention. It has also been suggested that the collection of unbiased, objective data is particularly important because of claims by the tobacco industry and others that the Scottish "smoke free" legislation would have negative economic and other effects [31].

A further element to be considered in weighing the balance of risks and benefits relates to the collection and dissemination of information on the extent to which bars do not comply with the legislation. If the rate of non-compliance were found to be high, and if this information were then to become widely available, then in theory this could lead to the imposition of sanctions against their owners (such as fines). In practice this information on compliance was already being collected on an official basis with Environmental Health Officers in Scotland being responsible for monitoring whether smokers were being permitted to smoke on the premises. The researchers were therefore collecting data which were already available to those responsible for enforcing the legislation. No significant additional risk to businesses was therefore posed by the research project.

Overall, we felt that the balance of risks and benefits was much in favour of collecting these data covertly, particularly as other options were not available to us. For example, whereas data on changes in air quality could perhaps have been obtained remotely by means of unattended air samplers, the collection of data on actual smoking behaviour (as opposed to the presence of smoke) required the presence of researchers. However, the balance of risks and benefits need to be considered explicitly in advance, and obviously they may be very different in different settings. In some, covert observation may be felt to contravene the rights of smokers to smoke unobserved, or the need to obtain informed consent may outweigh the need to collect unbiased data. However, we felt that the collection of anonymised data in public places with no intervention necessary from the researcher outweighed the risks, and the procedure was consistent with very many other studies which have unobtrusively collected data on human behaviours (including smoking) in public settings.

## Conclusion

These findings may be of value to researchers planning similar studies and to others who need to assess the effects of legislative or other interventions on health behaviours. Some of the objections to such studies relate to concerns about the safety of researchers, and the studies described above have found ways of mitigating potential risks. Overall, these observational data will play an important part in assessing the impact of the ban in different communities; this in turn will allow some assessment of whether such legislation can contribute to tackling smoking-related inequalities in health. The final outcome data will be available in summer 2007.

## Appendix: Members of the CLEAN Collaboration

NHS Health Scotland: Sally Haw and Laurence Gruer.

ISD Scotland: Colin Fischbacher and Diane Stockton.

Scottish Executive: Calum Scott.

CHETS: Candace Currie, Patricia Akhtar and Dorothy Currie, CAHRU, University of Edinburgh, Edinburgh.

HEPS: Sally Malam and Ruth Gosling, BMRB, London.

STOPIT: Jill Pell, University of Glasgow, Glasgow; Stuart Cobbe and John Rodgers, Glasgow Royal Infirmary, Glasgow; Frank Dunn, Anne Wright and Nat Hawkins, Stobhill Hospital, Glasgow; Timothy Gilbert and James Young, Hairmyres Hospital, East Kilbride; Paul MacIntyre and Jacqueline Dougall, Royal Alexandra Hospital, Helensburgh; David Murdoch and Anne Andrews, Southern General Hospital, Glasgow; David Newby and Sharon Cameron, Royal Infirmary Edinburgh; Keith Oldroyd, Joanne Kelly and Fiona Stevenson, Western Infirmary, Glasgow; Alastair Pell and Judith Anderson, Monklands Hospital, Lanarkshire; Stuart Pringle and Helen Marshall, Ninewells Hospital, Dundee.

BHETSE: Jonathon Ayres, Sean Semple and Anne Ludbrook, University of Aberdeen, Aberdeen; Fintan Hurley and Graeme Hughson, Institute of Occupational Medicine, Edinburgh; Shona Hilton, Mark Petticrew, MRC Social and Public Health Sciences Unit, Glasgow.

Qualitative Bar Study: Gerard Hastings, Douglas Eadie and Susan MacAskill, University of Stirling and the Open University, Stirling; John Davies, Derek Heim and Alastair Ross, University of Strathclyde, Glasgow.

Qualitative Community Study: Claudia Martin, Scottish Centre for Social Research, Edinburgh; Amanda Amos and Deborah Ritchie, University of Edinburgh, Edinburgh.

ITC Ireland/UK Scotland extension: Geoffrey T. Fong, Department of Psychology, University of Waterloo, Waterloo, Canada; Gerard Hastings and Louise Hassan, University of Stirling and the Open University, Stirling; Andy Hyland, Roswell Park Cancer Institute, New York.

## Competing interests

The author(s) declare that they have no competing interests.

## Authors' contributions

All authors participated in the design of their respective studies discussed in the workshop. MP drafted the manuscript. SS coordinated the workshop. SS, SH, KSC, DR participated directly in the covert observations discussed. All authors participated in the workshop, commented on drafts of the manuscript and approved the final manuscript.

**Table 2:** Key issues in covert observational research

1. Fieldworker safety is paramount; fieldworkers should be aware of when, and how to abandon data collection. Lone worker protocols are also important (see text).
2. Detailed data collection protocols are essential to limit potential bias
3. Training on data collection, preferably involving role playing and visits to the site where observation will take place, is essential
4. Observers should be matched to the environment, for example by age and gender
5. Working in pairs may help fieldworkers feel safer, and less conspicuous, and may limit biases in data collection (though this will increase research costs)
6. Despite all possible precautions, covert observation may be noticed and queried; fieldworkers should therefore have a plausible reason for being where they are

## Pre-publication history

The pre-publication history for this paper can be accessed here:


